# Supplementary material for: Cytomegalovirus reactivation under pre-emptive therapy following allogeneic hematopoietic stem cell transplant: Pattern, survival, and risk factors in the Republic of Korea
Source: PLoS One. 2023 Sep 13;18(9):e0291268. doi: 10.1371/journal.pone.0291268 (PMC10499250; doi:10.1371/journal.pone.0291268)
Supplement: S1 File — (DOCX) [file pone.0291268.s001.docx]

STROBE Statement—checklist of items that should be included in reports of observational studies

|  | Item No. | Recommendation | Page  No. | Relevant text from manuscript |
| --- | --- | --- | --- | --- |
| **Title and abstract** | 1 | (*a*) Indicate the study’s design with a commonly used term in the title or the abstract | 2 | Methods: We retrospectively analyzed the patterns and survival after CMV reactivation in patients undergoing pre-emptive therapy during allo-HSCT and assessed high-risk patients who could benefit from aggressive CMV prophylaxis in endemic areas. |
|  |  | (*b*) Provide in the abstract an informative and balanced summary of what was done and what was found | 2 | Conclusion: Despite half the allo-HSCT patients experiencing CMV reactivation, it did not affect survival, indicating that pre-emptive therapy has advantages, even in CMV-endemic areas. However, patients with multiple myeloma, CMV seropositivity of the recipient and donor, and those treated with high-dose ATG should be given aggressive CMV primary prophylaxis. Patients who experience CMV reactivation or aGVHD requiring steroid therapy should be considered for aggressive secondary prophylaxis. |
| Introduction | | | |  |
| Background/rationale | 2 | Explain the scientific background and rationale for the investigation being reported | 3 | - Cytomegalovirus (CMV) is endemic to the Republic of Korea.  - CMV primary prophylaxis is recommended in patients at high risk of CMV reactivation during allo-HSCT, with letermovir being considered aggressive primary prophylaxis in CMV-seropositive recipients.  - However, there are insufficient data on whether the conventional pre-emptive therapy for CMV has poor clinical outcomes in CMV-endemic areas and whether this pre-emptive strategy should be changed to more aggressive prophylaxis in all patients. |
| Objectives | 3 | State specific objectives, including any prespecified hypotheses | 4, 5 | In this study, we retrospectively analyzed the patterns and survival of CMV reactivation in patients undergoing pre-emptive therapy for CMV infection during HSCT and assessed high-risk patients who could benefit from aggressive primary or secondary CMV prophylaxis in a CMV-endemic area. |
| Methods | | | |  |
| Study design | 4 | Present key elements of study design early in the paper | 4, 5 | We retrospectively analyzed data from three tertiary hospitals affiliated with the Korea University Medical Center (Anam, Guro, and Ansan Hospitals) in the Korea University Transplant Registry from November 2003 to July 2020. |
| Setting | 5 | Describe the setting, locations, and relevant dates, including periods of recruitment, exposure, follow-up, and data collection | 4, 5 | (Same as above mentioned) We retrospectively analyzed data from three tertiary hospitals affiliated with the Korea University Medical Center (Anam, Guro, and Ansan Hospitals) in the Korea University Transplant Registry from November 2003 to July 2020. |
| Participants | 6 | (*a*) *Cohort study*—Give the eligibility criteria, and the sources and methods of selection of participants. Describe methods of follow-up  *Case-control study*—Give the eligibility criteria, and the sources and methods of case ascertainment and control selection. Give the rationale for the choice of cases and controls  *Cross-sectional study*—Give the eligibility criteria, and the sources and methods of selection of participants | 4, 5 | Patients who met all the following criteria were included: (1) had undergone allo-HSCT; (2) were monitored for CMV reactivation using CMV antigen or CMV polymerase chain reaction (PCR) during and for up to six months after the allo-HSCT; and (3) were treated with pre-emptive therapy for CMV reactivation. Patients in whom the first CMV reactivation was six months after the allo-HSCT, and those who received salvage treatment due to disease recurrence before the first CMV reactivation were excluded. To include recurrent CMV reactivation after the first CMV reactivation, all observable periods were checked. |
|  |  | (*b*) *Cohort study*—For matched studies, give matching criteria and number of exposed and unexposed  *Case-control study*—For matched studies, give matching criteria and the number of controls per case | N/A | N/A |
| Variables | 7 | Clearly define all outcomes, exposures, predictors, potential confounders, and effect modifiers. Give diagnostic criteria, if applicable | 6, 7 | The primary endpoints were the patterns and survival of patients with CMV reactivation who had undergone CMV monitoring and pre-emptive therapy for CMV infection during allo-HSCT in a CMV-endemic area. The secondary endpoint was the investigation of high-risk patients who could benefit from aggressive primary or secondary CMV prophylaxis. The following data were collected: age, sex, diagnosis, type of donor (matched, mismatched at one allele, or haploidentical), hematopoietic cell transplantation-specific comorbidity index (HCT-CI score), CMV serostatus of the donor and recipient, type of conditioning regimen (non-myeloablative, reduced intensity, or myeloablative), ATG use and dose, type of immunosuppressant (cyclosporine or tacrolimus), the occurrence of acute GVHD grade ≥2 or chronic GVHD with more than moderate severity, and recurrence of the disease. The occurrence of acute GVHD grade ≥2 or chronic GVHD with more than moderate severity was distinguished using an operational definition. According to the general guidelines of GVHD and treatment policy of the Korea University Medical Center, we had been treating patients with acute GVHD grade ≥2 or chronic GVHD with more than moderate severity using systemic steroid therapy with a dosage of at least 1 mg/kg of actual bodyweight or higher for at least 7 days. Therefore, we classified patients with acute GVHD grade ≥2 or chronic GVHD with more than moderate severity as those who received systemic steroid therapy with an aforementioned dosage. Acute GVHD was classified as patients who met the aforementioned criteria within 100 days from the transplantation date, while chronic GVHD was classified as patients who met the aforementioned criteria after 100 days. |
| Data sources/ measurement | 8* | For each variable of interest, give sources of data and details of methods of assessment (measurement). Describe comparability of assessment methods if there is more than one group | 7, 8 | Median values and ranges were reported for continuous variables, and percentages were reported for categorical values. Categorical values were analyzed using the chi-squared test. Overall survival (OS) was defined as the time from day 0 of allo-HSCT to death from any cause or censoring. Factors affecting OS or CMV reactivation were assessed using the Cox proportional hazards model for univariate and multivariate analyses. |
| Bias | 9 | Describe any efforts to address potential sources of bias | 7, 8 | OS was analyzed using the following variables: age, sex, diagnosis, type of donor, HCT-CI score, type of conditioning regimen, ATG use, type of immunosuppressant, CMV reactivation after allo-HSCT, the occurrence of acute GVHD grade ≥2 or chronic GVHD with more than moderate severity during all observable periods, and recurrence of the disease. The risk of the first CMV reactivation was analyzed using the following variables: age, sex, diagnosis, type of donor, HCT-CI score, CMV serostatus of the donor and recipient, type of conditioning regimen, ATG use and dose, type of immunosuppressant, the occurrence of acute GVHD grade ≥2, and the occurrence of chronic GVHD with more than moderate severity within six months after the date of allo-HSCT. |
| Study size | 10 | Explain how the study size was arrived at | 8 | We conducted a study on a total of 292 patients who met the inclusion criteria in the Korea University Transplant Registry. |

Continued on next page

| Quantitative variables | 11 | Explain how quantitative variables were handled in the analyses. If applicable, describe which groupings were chosen and why | 7 | Median values and ranges were reported for continuous variables, and percentages were reported for categorical values. Categorical values were analyzed using the chi-squared test. |
| --- | --- | --- | --- | --- |
| Statistical methods | 12 | (*a*) Describe all statistical methods, including those used to control for confounding | 7, 8 | Overall survival (OS) was defined as the time from day 0 of allo-HSCT to death from any cause or censoring. Factors affecting OS or CMV reactivation were assessed using the Cox proportional hazards model for univariate and multivariate analyses. OS was analyzed using the following variables: age, sex, diagnosis, type of donor, HCT-CI score, type of conditioning regimen, ATG use, type of immunosuppressant, CMV reactivation after allo-HSCT, the occurrence of acute GVHD grade ≥2 or chronic GVHD with more than moderate severity during all observable periods, and recurrence of the disease. The risk of the first CMV reactivation was analyzed using the following variables: age, sex, diagnosis, type of donor, HCT-CI score, CMV serostatus of the donor and recipient, type of conditioning regimen, ATG use and dose, type of immunosuppressant, the occurrence of acute GVHD grade ≥2, and the occurrence of chronic GVHD with more than moderate severity within six months after the date of allo-HSCT. IBM SPSS Statistics for Windows, version 21.0 (IBM Corp., Armonk, NY, USA) was used for data analysis. P <0.05 was considered statistically significant. |
|  |  | (*b*) Describe any methods used to examine subgroups and interactions | N/A | N/A |
|  |  | (*c*) Explain how missing data were addressed |  | This study analyzed patients within the Korea University Transplant Registry who belonged to the inclusion criteria, and there were no missing values in the necessary variables for the analysis. |
|  |  | (*d*) *Cohort study*—If applicable, explain how loss to follow-up was addressed  *Case-control study*—If applicable, explain how matching of cases and controls was addressed  *Cross-sectional study*—If applicable, describe analytical methods taking account of sampling strategy | N/A | N/A |
|  |  | (*e*) Describe any sensitivity analyses | 8 | IBM SPSS Statistics for Windows, version 21.0 (IBM Corp., Armonk, NY, USA) was used for data analysis. P <0.05 was considered statistically significant. |
| Results | | | | |
| Participants | 13* | (a) Report numbers of individuals at each stage of study—eg numbers potentially eligible, examined for eligibility, confirmed eligible, included in the study, completing follow-up, and analysed | 8 | A total of 292 patients who underwent allo-HSCT were analyzed (Table 1). |
|  |  | (b) Give reasons for non-participation at each stage | N/A | N/A |
|  |  | (c) Consider use of a flow diagram | N/A | This study analyzed patients within the Korea University Transplant Registry who met the inclusion criteria, and it was determined that the benefits of adding a flow diagram were not significant, so one was not created. |
| Descriptive data | 14* | (a) Give characteristics of study participants (eg demographic, clinical, social) and information on exposures and potential confounders | 8, 9, 10 | A total of 292 patients who underwent allo-HSCT were analyzed (Table 1). |
|  |  | (b) Indicate number of participants with missing data for each variable of interest | N/A | N/A |
|  |  | (c) *Cohort study*—Summarise follow-up time (eg, average and total amount) | 4 | Patients in whom the first CMV reactivation was six months after the allo-HSCT, and those who received salvage treatment due to disease recurrence before the first CMV reactivation were excluded. To include recurrent CMV reactivation after the first CMV reactivation, all observable periods were checked. |
| Outcome data | 15* | *Cohort study*—Report numbers of outcome events or summary measures over time | 8-15 | - Pattern and survival of CMV reactivation in patients undergoing CMV monitoring and pre-emptive therapy for CMV infection during allo-HSCT (Figure 1, and Table 2)  - Patients at high risk for CMV reactivation (Figure 2, and Table 3) |
|  |  | *Case-control study—*Report numbers in each exposure category, or summary measures of exposure | N/A | N/A |
|  |  | *Cross-sectional study—*Report numbers of outcome events or summary measures | N/A | N/A |
| Main results | 16 | (*a*) Give unadjusted estimates and, if applicable, confounder-adjusted estimates and their precision (eg, 95% confidence interval). Make clear which confounders were adjusted for and why they were included | 12-15 | Table 2, and 3 |
|  |  | (*b*) Report category boundaries when continuous variables were categorized | 9-11, and 12-15 | Table 1, 2, and 3 |
|  |  | (*c*) If relevant, consider translating estimates of relative risk into absolute risk for a meaningful time period | 12-15 | Table 2, and 3 |

Continued on next page

| Other analyses | 17 | Report other analyses done—eg analyses of subgroups and interactions, and sensitivity analyses | N/A | N/A |
| --- | --- | --- | --- | --- |
| Discussion | | | | |
| Key results | 18 | Summarise key results with reference to study objectives | 16 | In this study, 47.6% of the patients showed CMV reactivation and were treated with pre-emptive therapy during allo-HSCT. CMV reactivation and treatment with pre-emptive therapy did not affect the survival of these patients. However, approximately half of these patients experienced CMV reactivation more than twice, with a median treatment duration of 10‒15 days. The risk of CMV reactivation was higher in patients with multiple myeloma, CMV seropositivity of recipients and donors before allo-HSCT, acute GVHD grade ≥2, and the use and dose of ATG. |
| Limitations | 19 | Discuss limitations of the study, taking into account sources of potential bias or imprecision. Discuss both direction and magnitude of any potential bias | 17, 18 | This study had several limitations. First, this study retrospectively analyzed data from a small number of patients. We aimed to present the pattern and effect of CMV reactivation on clinical outcomes in CMV-endemic areas; however, the results of this study did not represent the entire situation in CMV-endemic areas. In addition, although all patients in this study underwent allo-HSCT, they had various diseases, including aplastic anemia, myelodysplastic syndrome, leukemia, multiple myeloma, and lymphoma. Before allo-HSCT, each patient was treated with different types and durations of chemotherapy or immunosuppressive therapies. Therefore, this study alone cannot confirm the above conclusions, and additional studies are required to accurately classify the risk of CMV reactivation in each patient group. Third, we used operational definitions when defining the factors of acute or chronic GVHD that could potentially influence the interpretation of clinical endpoints results. In clinical practice, GVHD diagnosis is not solely based on objective indicators. Therefore, the operational definition of patients who received systemic steroid therapy at a dosage of 1 mg/kg of actual body weight or higher for a minimum of 7 days or more is not considered inappropriate. However, patients with mild GVHD might have been included, and the possibility of misdiagnosis of organ involvement as GVHD due to other causes, such as CMV infections, cannot be completely ruled out. Although we controlled for each factor through multivariate analysis, the results should be interpreted within the limitations of the retrospective study design. |
| Interpretation | 20 | Give a cautious overall interpretation of results considering objectives, limitations, multiplicity of analyses, results from similar studies, and other relevant evidence | 16 | Although the incidence of CMV reactivation can differ according to the situation at the time of transplantation, region, and monitoring methods for CMV, reactivation has been reported to develop in approximately 50% of cases (range: 10–70%) 4–8 weeks after allo-HSCT. In this study, 47.6% of patients showed CMV reactivation, most of which occurred within 100 days (median: 31.5 days, range: 29.0‒180.0 days). CMV is endemic in the Republic of Korea; however, the incidence of CMV reactivation was comparable to previously reported values. |
| Generalisability | 21 | Discuss the generalisability (external validity) of the study results | 18 | Nevertheless, it is meaningful that this study presented the real-world pattern and survival in patients undergoing CMV monitoring and pre-emptive therapy for CMV reactivation during HSCT in a CMV-endemic area and suggested that high-risk patients can benefit from more aggressive CMV prophylaxis. This study presented real-world data that could serve as a basis for broadening the indication of primary prophylaxis for CMV reactivation and its use as secondary prophylaxis. |
| Other information | |  | | |
| Funding | 22 | Give the source of funding and the role of the funders for the present study and, if applicable, for the original study on which the present article is based | 19 | This research was supported by a Korea University Grant (number: K2125711). The funders had no role in study design, data collection and analysis, decision to publish, or preparation of the manuscript. |

*Give information separately for cases and controls in case-control studies and, if applicable, for exposed and unexposed groups in cohort and cross-sectional studies.

**Note:** An Explanation and Elaboration article discusses each checklist item and gives methodological background and published examples of transparent reporting. The STROBE checklist is best used in conjunction with this article (freely available on the Web sites of PLoS Medicine at http://www.plosmedicine.org/, Annals of Internal Medicine at http://www.annals.org/, and Epidemiology at http://www.epidem.com/). Information on the STROBE Initiative is available at www.strobe-statement.org.
