# Supplementary material for: Cytomegalovirus reactivation under pre-emptive therapy following allogeneic hematopoietic stem cell transplant: Pattern, survival, and risk factors in the Republic of Korea
Source: PLoS One. 2023 Sep 13;18(9):e0291268. doi: 10.1371/journal.pone.0291268 (PMC10499250; doi:10.1371/journal.pone.0291268)
Supplement: Attachment — Submitted filename: PLOS ONE-PONE-D-23-13734.doc [file pone.0291268.s003.doc]

**Title:** Cytomegalovirus reactivation under pre-emptive therapy during allogeneic hematopoietic stem cell transplant: pattern, survival, and risk factors in the Republic of Korea

**Comments to the Author**

In their manuscript Dr Kang and colleagues retrospectively analyzed the patterns and survival after CMV reactivation in patients undergoing pre-emptive therapy during allo-HSCT in CMV-endemic areas. They also assessed patients at high risk for CMV reactivation, and suggested that those patients might benefit from aggressive CMV prophylaxis. Their work has some significance, but further analysis of the data is needed, and the structure and language expression of the article need to be further modified, and some errors need to be corrected.

**Major comments:**

Q1: In page 2, the manuscript mentioned that “Pre-emptive therapy for cytomegalovirus (CMV) reactivation has been used in allogeneic hematopoietic stem cell transplant (allo-HSCT). It is unclear if this strategy has poorer clinical outcomes in CMV-endemic areas and if more aggressive prophylaxis should be needed”. The overall survival of patients with CMV reactivation was compared with those without reactivation. I think it is CMV reactivation that has poor clinical outcomes, but not “Pre-emptive therapy for CMV”. Please modify the language expression.

Q2: The overall survival in the subgroup of high-risk patients were not analyze. Besideds OS, treatment related mortality, CMV associated mortality should also be analyzed. It is better to add these analysis to further concluded that those hihg-risk patients might benefit from aggressive CMV prophylaxis.

Q3: In page 5, the manuscript mentioned that “Patients in whom the first CMV reactivation was six months after the allo-HSCT”, what dose it mean?

Q4: Detail treatment for CMV should be described. Only ganciclovir were used for CMV? Were any of the enrolled 292 patients developed refractory CMV infection or CMV disease? According to the publications (Pei XY, Huang XJ et al., JID 2017, CID 2019, CMI 2022), the application of adoptive CMV-specific T-cell immunotherapy has been attempted for refractory CMV infection in China. Were any of the 292 aptients received DLI, CMV-specific T cell adoptive immunotherapy, or CMV specific immunoglobulin?

Q5: Were any of the enrolled patients developed EBV? Whta was anti-EBV therapy (especially CD20 monoclonal antibody)?

Q6: The donor type is not clear. As in text and in Table 1-4, what is the difference between “mismatched at one allele” and “haploidentical”? Dose “mismatched at one allele” not incleded in “haploidentical”? Or you mean “mismatched at one allele” were from unrealted donors?

**Minor comments:**

Q6: Correct mistake in Table 1 “Female, n(%), 1294 (44.2%)”.

Q7: There are many repeated statements in the discussion, please streamline the language
