# Supplementary material for: Cytomegalovirus reactivation under pre-emptive therapy following allogeneic hematopoietic stem cell transplant: Pattern, survival, and risk factors in the Republic of Korea
Source: PLoS One. 2023 Sep 13;18(9):e0291268. doi: 10.1371/journal.pone.0291268 (PMC10499250; doi:10.1371/journal.pone.0291268)
Supplement: Attachment — Submitted filename: 0_Response_to_reviewers_20230725.docx [file pone.0291268.s004.docx]

**Manuscript ID: PONE-D-23-13734**

**Title:** Cytomegalovirus reactivation under pre-emptive therapy following allogeneic hematopoietic stem cell transplant: pattern, survival, and risk factors in the Republic of Korea

Dear Prof. George Vousden:

Thank you for your help in processing our manuscript, and we appreciate the reviewers’ thoughtful comments. First, we have formatted the manuscript according to the style requirements of PLOS ONE and added or modified content regarding participant consent and the Data Availability statement. We have also revised the manuscript in accordance with the reviewers’ comments and suggestions. Point-by-point responses are included in this letter to each comment of the reviewers. Our revisions address all points raised by the reviewers. Additionally, our manuscript has been reviewed by an English-speaking editor.

We believe that changes made in response to the reviewers’ comments have significantly improved the revised manuscript, and we hope that the revised manuscript is now suitable for publication.

Sincerely yours,

Hwa Jung Sung, MD, PhD

Department of Internal Medicine, Korea University College of Medicine 73 Goryeodae-ro, Seongbuk-gu, Seoul 02841, Republic of Korea

E-mail: [doctorsung@korea.ac.kr](mailto:doctorsung@korea.ac.kr); Telephone: +82-31-412-4938; Fax: +82-31-412-5802

**List of reviewers’ comments and point-by-point responses**

**In each response, page and line numbers refer to the revised manuscript.**

**In the revised manuscript, text changes are highlighted in yellow.**

**Reviewer #1:**

In their manuscript Dr Kang and colleagues retrospectively analyzed the patterns and survival after CMV reactivation in patients undergoing pre-emptive therapy during allo-HSCT in CMV-endemic areas. They also assessed patients at high risk for CMV reactivation, and suggested that those patients might benefit from aggressive CMV prophylaxis. Their work has some significance, but further analysis of the data is needed, and the structure and language expression of the article need to be further modified, and some errors need to be corrected.

**1. Abstract:**

**Introduction: should be transplantation not transplant**

**Methods: post-HSCT not during HSCT**

**Conclusions duplicate the results and should be corrected.**

***Response***: Thank you for your careful review of our manuscript. As you suggested, we have modified this (page 2, lines 16, 19, and 30–33). In addition, following your recommendation, we have changed the title of the manuscript from “Cytomegalovirus reactivation under pre-emptive therapy during allogeneic hematopoietic stem cell transplant: pattern, survival, and risk factors in the Republic of Korea” to “Cytomegalovirus reactivation under pre-emptive therapy following allogeneic hematopoietic stem cell transplant: pattern, survival, and risk factors in the Republic of Korea.” We have also made necessary modifications to the related words in the manuscript.

**2. Introduction:**

**(1) Recipients should be added that it concerns stem cell recipients**

***Response***: Thank you for your careful review of our manuscript. As you pointed out, we have modified this (page 3, lines 43 and 47).

**(2) CMV seropositive recipients have higher risk of CMV seronegative recipients only if blood products are CMV seronegative**

***Response***: Thank you for your careful review of our manuscript. As you suggested, we have modified this (page 3, lines 42–46).

**(3) Please clarify the term “aggressive” prophylaxis used in the context of letermovir prophylaxis (as this term does not appear in the literature) referring to a reliable source or replace with “primary prophylaxis”.**

***Response***: Thank you for your careful review of our manuscript. We have modified the sentence from “CMV primary prophylaxis is recommended in patients at high risk of CMV reactivation during allo-HSCT, with letermovir being considered aggressive primary prophylaxis in CMV-seropositive recipients” to “CMV primary prophylaxis is recommended in recipients of allo-HSCT at high risk of CMV reactivation following allo-HSCT, with letermovir being considered in CMV-seropositive recipients.” (page 3, lines 48–50).

**3. Methods:**

**(1) It is not clear from this section whether the authors use letermovir in CMV primary prophylaxis; in introduction the authors mentioned that LMV should be used in almost all Korean stem cell recipients.**

***Response***: Thank you for the suggestion. In the Republic of Korea, letermovir has been covered by national insurance since August 2020. When we identified patients who met the indications for letermovir usage, most were recommended its use because the Republic of Korea is a CMV-endemic area. Therefore, we aimed to obtain objective information to identify the advantages and disadvantages of existing CMV pre-emptive therapies and why letermovir should be used in asymptomatic high-risk patients in this study. Consequently, patients who received letermovir as CMV primary prophylaxis were excluded from this study. The manuscript has been revised to ensure that this information is conveyed in the main text (page 3, line 58–59, page 4, lines 60, 62–63, and 73–76).

**(2) CMV monitoring weekly or twice weekly, but how long? To day +100 or longer?**

***Response***: Thank you for your careful review of our manuscript. We included only patients who had been monitored for CMV reactivation using the CMV antigen or CMV PCR for at least 6 months from the date of allo-HSCT. The manuscript has been revised to convey this information (page 4, lines 71–73, and page 6, lines 113–114).

**(3) Ganciclovir was used untill CMV was undetectable; does it mean that it could be a continuous therapy?; what about an oral agent (valganciclovir) which is commonly used in clinical practice?**

***Response***: Thank you for your careful review of our manuscript. Pre-emptive therapy for CMV was continuously administered with ganciclovir (5 mg/kg of actual body weight, through intravenous injection every 12 h) until a negative test result for the CMV antigen or a CMV PCR titer of <1,000 copies/mL was noted. The oral agent valganciclovir is available for use in the Republic of Korea but with insurance coverage issues in the study period. At our medical center, pre-emptive therapy for CMV was administered using the intravenous agent ganciclovir for all patients requiring pre-emptive therapy for CMV according to the treatment policy at the Korea University Medical Center. The manuscript has been revised to convey this information (page 6, lines 117–121).

**(4) ATG horse or rabbit? For whom? For matched related recipients too?**

***Response***: Thank you for the suggestion. In the Republic of Korea, only antithymocyte globulin rabbits are available. The manuscript has been revised to convey this information (page 5, lines 106–107, and page 6, lines 108–109).

We retrospectively analyzed data from three tertiary hospitals affiliated with the Korea University Medical Center (Anam, Guro, and Ansan) in the Korea University Transplant Registry. Rabbit anti-thymocyte globulin was used at the discretion of the participating physicians, depending on the type of donor and conditioning regimen. Reviewing the patients analyzed in this study revealed that even among matched related recipients, some received rabbit anti-thymocyte globulin (57/200 patients).

**(5) Information provided in the last sentence of the first paragraph (“We accessed the data from January 2022 to December 2022 for the purpose of data collection and research”) is irrelevant.**

***Response***: Thank you for your careful review of our manuscript. This content was requested by the editorial office during the initial submission of the manuscript. We have removed this information from the manuscript and moved the relevant information to an appropriate section.

**4. Results:**

**(1) Did the authors observe any CMV diseases in CMV reactivated patients? What includes the term “infections” as a cause of death.**

***Response***: Thank you for the suggestion. Infections were calculated as the mean of all forms of infection (bacterial, fungal, viral, etc.). Patients who died of infections were administered broad-spectrum antibiotics and antifungal agents until the day of death, making it difficult to conclusively attribute their deaths to CMV disease in such circumstances. Therefore, we presented patients who died from all forms of infection and the number of patients who received pre-emptive therapy for CMV reactivation using ganciclovir up to 1 week before their death. As you pointed out, we have revised the manuscript accordingly (page 11, lines 194–198).

**(2) In patients with intestinal acute GVHD did the authors check for CMV colitis?**

***Response***: Thank you for the suggestion. In this study, the occurrence of acute GVHD grade ≥2 or chronic GVHD with more than moderate severity was distinguished using an operational definition: patients who received systemic steroid therapy at a dosage of 1 mg/kg of actual body weight or higher for a minimum of 7 days or more. (We describe the details of the contents corresponding to the following (5) questions below). This was done to address missing values resulting from patient compliance issues (including inaccurate measurement of diarrhea quantity), the inability to conduct appropriate tests owing to a deteriorating condition, or insufficient information in the medical chart. However, in clinical practice, GVHD is not determined solely based on objective indicators. Therefore, although classifying patients based on systemic steroid use has limitations, it is considered appropriate.

As you pointed out, distinguishing whether this is because of acute GVHD or CMV enteritis in patients with gastrointestinal problems is necessary to interpret the research results accurately. To verify this situation based on objective evidence, patients with acute GVHD who underwent upper or lower gastrointestinal endoscopy were examined, and patients with histologically confirmed CMV colitis were identified. Of the 292 patients, 99 (33.9%) experienced acute GVHD grade ≥2, of which 25 underwent upper gastrointestinal endoscopy and 30 underwent lower gastrointestinal endoscopy. In four patients, CMV enteritis was confirmed by pathological examination. However, all four patients simultaneously received treatment for both acute GVHD with systemic steroids and CMV reactivation during this period. Therefore, the exact causes of the gastrointestinal problems experienced by the patients could not be established. The overall impact on the results would be minimal because the number of relevant patients was small, and the main clinical endpoints underwent a multivariate analysis with adjustments for these factors. However, the potential limitations of this approach have been mentioned in the Discussion section (page 18, lines 290–299).

**(3) Max duration of first anti-CMV treatment was 73 days; does it mean that some patients were receiving ganciclovir for more than 2 months?**

***Response***: Thank you for your careful review of our manuscript. The original dataset confirmed that one of the 139 patients who received the first pre-emptive therapy for CMV received treatment for 73 days. This is considered an extraordinary case. However, since the patient's data had no problems, we have presented the case without deleting it.

**(4) Table 2 only statistically significant values should be provided.**

***Response***: Thank you for the suggestion. We have made the necessary modifications to **Table 2** according to your recommendations.

**(5) I haven’t found information on how many patients developed acute GVHD, stage? Organ involvement? Treatment? It is crucial for CMV reactivation.**

***Response***: Thank you for the suggestion. This study was based on a retrospective chart review. Initially, we attempted to distinguish between patients who experienced acute GVHD grade ≥2 or chronic GVHD with more than moderate severity using data from medical charts according to existing definitions. However, this was difficult because of missing data. Therefore, we decided to switch the approach to distinguish patients with acute GVHD grade ≥2 or chronic GVHD of more than moderate severity. We have been treating patients with acute GVHD grade ≥2 or chronic GVHD with more than moderate severity using systemic steroid therapy with a dosage of at least 1 mg/kg of actual body weight or higher for more than a minimum of 7 days, according to the general guidelines of GVHD and treatment policy at the Korea University Medical Center. Therefore, based on the treatment policy of the Korea University Medical Center, we classified patients with acute GVHD grade ≥2 or chronic GVHD with more than moderate severity as those who received systemic steroid therapy at a dosage of at least 1 mg/kg of actual body weight or higher for more than 7 days.

- To clearly express this aspect, we have revised the relevant content in the Methods section (page 7, lines 133–143).
- The number of patients with acute GVHD grade ≥2 or chronic GVHD of more than moderate severity is shown in **Table 1**. Additionally, relevant information has been included in the Results section of the manuscript (page 8, lines 174–176).
- In the Discussion section, we have mentioned the limitations arising from distinguishing patients with acute GVHD grade ≥2 or chronic GVHD with more than moderate severity using an operational definition (page 18, lines 290–299).

**(6) Table 3 should be removed, no important information included.**

***Response***: Thank you for the suggestion. We have deleted **Table 3** and adjusted the manuscript content accordingly.

**(7) Table 4 only statistically significant data should be left.**

***Response***: Thank you for the suggestion. We have made necessary modifications to **Table 4** according to your recommendations. As Table 3 has been deleted, the table number has been changed from #4 to #3.

**(8) Table 5 to be removed.**

***Response***: Thank you for the suggestion. We have deleted **Table 5** and adjusted the manuscript content accordingly.

**(9) Did the authors still use the same anti-viral agent even when there was a repeated CMV reactivation? No one received foscavir or cidofovir?**

***Response***: Thank you for the suggestion. In the Republic of Korea, foscavirs face cost-related issues, as they are not covered by insurance for CMV reactivation. However, cidofovir is available as a rare medicine only through the Korea Orphan & Essential Drug Center, which makes its distribution challenging. In this study, of 139 patients who experienced CMV reactivation, three received foscarnet for uncontrolled CMV reactivation, with no users of cidofovir. The reason why the number of patients who used these medications was limited is uncertain between the effective control of CMV reactivation with ganciclovir alone and cost or distribution issues preventing their use. Therefore, we did not describe the relevant information in the manuscript, as it could exaggerate the effect of pre-emptive therapy for CMV reactivation.
